# Autonomization of Microvascular Free Flaps in Reconstructive Surgery: A Narrative Review

**DOI:** 10.1002/micr.70186

**Published:** 2026-01-30

**Authors:** Jonas Wüster, Leonard Knoedler, Tobias Niederegger, Leonard Simon Brandenburg, Gabriel Hundeshagen, Max Heiland, Steffen Koerdt

**Affiliations:** ^1^ Department of Oral and Maxillofacial Surgery, University Medical Center Freiburg, Faculty of Medicine Albert Ludwig University of Freiburg Berlin Germany; ^2^ Department of Oral and Maxillofacial Surgery Charité – Universitätsmedizin Berlin, Corporate Member of Freie Universität Berlin and Humboldt‐Universität Zu Berlin Berlin Germany; ^3^ Department of Hand, Plastic and Reconstructive Surgery Burn Trauma Center, BG Trauma Center Ludwigshafen, University of Heidelberg Ludwigshafen Germany

**Keywords:** flap autonomization, head and neck reconstruction, microvascular free flap, neovascularization, reconstructive surgery

## Abstract

**Background:**

Microvascular free tissue transfer is a key technique in reconstructive surgery, enabling functional and aesthetic restoration of complex defects. While initial flap survival relies on the vascular pedicle, some flaps may become independent through a process known as autonomization, where new vascular connections form between the flap and recipient site. Understanding the timeline, mechanisms, and clinical relevance of this process is essential for safe surgical planning and postoperative interventions.

**Methods:**

A narrative review was conducted to synthesize current literature on microvascular flap autonomization. Databases including PubMed and Google Scholar were searched up to June 2025, focusing on studies examining flap selection, neovascularization, perfusion monitoring, and predictors of flap vascular independence. Articles were screened based on relevance, methodological quality, and clinical applicability.

**Results:**

Flap autonomization showed heterogeneous timelines in literature. Skin and muscle flaps generally tolerated earlier pedicle compromise than jejunal or osteocutaneous flaps, while tissue composition, vascular contact area, recipient bed quality, and comorbidities strongly influenced revascularization. Favorable conditions—such as thin fasciocutaneous or muscle flaps on well‐perfused beds—were associated with earlier integration, whereas irradiated tissue and systemic vascular disease delayed independence. Monitoring tools (ICG angiography, laser Doppler, NIRS) aided perfusion assessment but could not confirm full autonomization. Complications were linked to delayed or incomplete neovascularization, particularly during secondary procedures. Adjunctive strategies, including ischemic conditioning and flap “training,” showed potential to promote vascular remodeling, but clinical evidence remains limited.

**Conclusion:**

Flap autonomization is a critical but poorly understood process that varies by flap type and patient context. Despite early neovascular activity, the lack of reliable markers necessitates conservative postoperative protocols. Emerging technologies and bioengineered strategies hold promise but require further validation. Standardized criteria to assess vascular independence could significantly improve outcomes in microvascular reconstructive surgery.

## 1 Introduction

Microvascular free flap surgery is a fundamental technique in modern reconstructive surgery, enabling the restoration of complex defects across a range of anatomical regions—including the head and neck, breast, trunk, extremities, and perineum—using well‐vascularized autologous tissue. Since its clinical inception in the early 1970s, the field has expanded significantly, driven by advances in microsurgical technique, flap origin, design, and perioperative care (Seidenberg et al. 1959; Antia and Buch 1971).

The growing repertoire of free flaps now includes musculocutaneous flaps (e.g., latissimus dorsi (LD)), fasciocutaneous flaps (e.g., radial free forearm (RFF), parascapular free flap (PSFF)), osteocutaneous flaps (e.g., fibula free flap (FFF)), and, as a more recent development, perforator‐based flaps (e.g., anterolateral thigh (ALT), deep inferior epigastric perforator (Chaya et al. 2021)), each offering specific advantages tailored to the defect's location, functional requirements, and donor site morbidity (Kruse et al. 2011; Engel et al. 2008; Wei et al. 2002; O'Connell et al. 2017; Wolff 2017). These options are routinely applied across diverse specialties, including breast reconstruction, limb salvage, perineal reconstruction, and head and neck surgery (Kim et al. 2020; Knoedler, Hoch, et al. 2024, 2025; Mark et al. 2022).

A central consideration in free flap surgery is not only long‐term viability but also the process of flap autonomization—the shift from reliance on the vascular pedicle to neovascularization from the recipient bed. From a reconstructive standpoint, this transition is particularly relevant when secondary procedures beneath or through the flap become necessary, as is common in orthoplastic surgery for delayed arthrodesis, hardware removal, or flap contouring. In these settings, a more randomized perfusion pattern provides greater procedural safety and flexibility, while the degree of autonomization limits the safe time interval before undertaking such secondary steps. The available literature offers heterogeneous and inconclusive reports on the timing of this transition, underscoring the need for further research (Sadove and Kanter 1993; Yoon and Jones 2016).

Therefore, this review aims to investigate the factors influencing autonomization of microvascular free flaps in reconstructive surgery, with emphasis on flap selection, perioperative planning, and the underlying mechanisms and clinical implications of this process. By synthesizing current evidence, it might support clinicians in optimizing reconstructive strategies and improving outcomes in free flap surgery.

## 2 Methods

This narrative review was undertaken to synthesize current literature on microvascular free tissue transfer and the process of flap autonomization in reconstructive surgery. The focus was placed on surgical techniques, flap selection, postoperative management, and the mechanisms underlying vascular independence. A structured yet adaptable methodology was employed to ensure a comprehensive and coherent analysis. The review process was informed by established guidance for narrative reviews, incorporating elements from the PRISMA 2020 framework and methodological principles recommended by Green et al. to ensure clarity, transparency, and academic rigor (Green et al. 2006).

### 2.1 Search Strategy

A comprehensive literature search was conducted using PubMed and Google Scholar up to June 2025 to identify relevant studies related to microvascular free tissue transfer and flap autonomization in reconstructive surgery. The search was designed to capture a wide range of relevant publications by employing a combination of thematic keywords and Boolean operators (AND, OR) to refine results. Key search terms included phrases related to microsurgical reconstruction (“microvascular free flap,” “free tissue transfer,” “reconstructive surgery”), vascularization processes (“neovascularization,” “angiogenesis,” “flap autonomization”), and clinical outcomes (“flap survival,” “pedicle failure,” “postoperative perfusion”). No limits were set regarding publication year, study design, or journal scope to ensure a thorough and inclusive review. Additionally, reference lists from foundational and frequently cited articles were manually screened.

### 2.2 Inclusion and Exclusion Criteria

This review included peer‐reviewed original studies, clinical trials (prospective and retrospective), systematic reviews, and meta‐analyses published up to May 2025 that addressed key aspects of microvascular free tissue transfer and flap autonomization. Eligible publications met at least one of the following criteria: (i) studies examining the surgical use, planning, or outcomes of microvascular free flaps in reconstructive procedures; (ii) research evaluating the biological mechanisms or clinical indicators of flap neovascularization and autonomization; (iii) investigations into risk factors for flap failure or complications related to vascular compromise; and (iv) reviews that synthesized evidence on flap selection, perfusion monitoring, or postoperative flap viability.

Exclusion criteria applied to studies that: (i) did not pertain to microvascular reconstructive surgery or vascularized flap procedures; (ii) were focused on unrelated surgical disciplines without crossover relevance; (iii) duplicated existing data without offering new insights; or (iv) lacked peer review or adequate methodological transparency for critical appraisal.

### 2.3 Data Extraction and Synthesis

Two authors independently reviewed all retrieved articles. Full‐text assessments were subsequently performed to evaluate each study's relevance to the objectives of this review. Inclusion was based on whether the study provided data or insights on flap selection, surgical technique, neovascularization processes, or postoperative flap viability. Any disagreements during the selection process were resolved through consensus discussion with a third reviewer, ensuring consistent and unbiased data inclusion. Key findings from the included studies were then thematically synthesized to highlight prevailing evidence, trends, and gaps in the literature.

## 3 Results

### 3.1 Timeline and Mechanisms of Flap Autonomization

Flap autonomization describes the process by which free flaps develop an independent blood supply through neovascularization, allowing survival without the original pedicle. The concept originated from clinical cases of unexpected flap survival after pedicle failure, prompting investigation into secondary perfusion mechanisms (Granzow et al. 2015; Chubb et al. 2010; Bradshaw and Wagels 2017; Rothaus and Acland 1983). Autonomization involves two key biological processes: angiogenesis, with capillary sprouting from the recipient bed beginning as early as postoperative day 3, and inosculation, where these new vessels connect with those in the flap. Studies suggest that skin and muscle flaps may tolerate vascular compromise earlier than jejunal or osteocutaneous flaps, while conservative therapies such as anticoagulation or leeching appear to facilitate survival after earlier occlusion. Rather than a fixed time window, autonomization should therefore be regarded as a gradual and context‐dependent process, with reported survival possible after pedicle loss in as little as a few days but more reliably beyond one to two postoperative weeks. Clinically, this uncertainty underpins why free flaps are often regarded as pedicle‐dependent for several weeks before performing secondary procedures across or beneath them (Yoon and Jones 2016; Berkane et al. 2023; Kaiser and Levin 2023; Patial et al. 2022; Singla et al. 2024; Rook et al. 2025). Table 1 presents an overview of free flap autonomization, including the relevant time frame and key landmarks.

**TABLE 1:** Overview of free flap autonomization time frame and key landmarks.

Time frame	Physiological phase	Pedicle dependence	Biological mechanism	Clinical dependency and status
Days 0–2	Plasmatic imbibition (Nelissen and White 2014; Smahel 1971; Stell 1977)	High dependency	No true blood flow between bed and flap. The flap absorbs nutrients via diffusion from the plasma layer (fibrin clot) formed between the flap and the recipient bed. Capillary permeability increases, causing edema.	100% pedicle dependent. The flap relies entirely on the microvascular anastomosis. Any compression or thrombosis results in total flap loss. Flap is maximally edematous.
Days 3–7	Inosculation (Yoon and Jones 2016; Angelos et al. 2010; Tsur et al. 1980; Foerster et al. 2022)	High dependency	“Osculum” = Latin for kiss. The cut ends of capillaries in the flap align with capillary buds sprouting from the recipient bed. A loose fibrin meshwork connects them. Histologically, there is anastomosis between existing vessels, not new vessel formation yet.	Critical Vulnerability. While connections exist, they are fragile. Shear forces (patient movement, hematoma) can tear these microscopic alignments. The flap is still fully dependent on the pedicle.
Days 7–14	Angiogenesis (neovascularization) (Godden and Thomas 2002; Wise et al. 2011; Burns et al. 2005; Yoon et al. 2018; Lucas 2017)	Moderate dependency	True ingrowth of new vessels (capillary loops) from the recipient bed into the flap, driven by hypoxia‐induced VEGF. Blood begins to flow from the bed into the flap's periphery. Capillary density increases.	Partial Independence. The periphery of the flap might survive pedicle failure, but the central bulk will necrose. Flap edema peaks around Day 5–7, then begins to subside as lymphatics reconnect.
Days 14–21	Vascular maturation (Berkane et al. 2023; Alsina‐Rius et al. 2022; Garutti et al. 2024)	Moderate dependency	The capillary loops differentiate. Smooth muscle cells (pericytes) coat the vessels, turning them into arterioles and venules capable of regulating flow (vasomotion). Vessel walls thicken. Collagen deposition begins.	Transitional phase. In healthy beds, the flap can often survive pedicle division. This is the optimal time to begin “flap training” (intermittent clamping of pedicle for 15 min/day) for staged pedicled flaps. Not functionally independent yet (Pignatti et al. 2012).
Weeks 3–4	Autonomization (Granzow et al. 2015; Mücke et al. 2012; Giordano et al. 2022)	Low dependency	The new vascular network is established and organized. Directional flow is stable (arterioles carry oxygenated blood into the flap; venules drain deoxygenated blood out). Neuronal ingrowth begins at the margins (return of protective sensation).	Functional Independence. The flap is generally considered independent of the main pedicle. Standard time for division of cross‐leg flaps or tube pedicles. This is the clinical “safe zone” for pedicle sacrifice in healthy beds.
6 weeks+	Long‐term remodeling (Bagheri et al. 2024; Ludolph et al. 2019; Thariat et al. 2024; Clarke and Chen 1992)	Low to independent	Regression of superfluous vessels (pruning). Alignment of collagen fibers along lines of mechanical stress. Lymphatic connections are re‐established (slowest process—can take 3–6 months). Flap becomes metabolically similar to native tissue.	Normalization. The flap physiology mimics the recipient site. In radiated or infected beds, this is the earliest safe point for independence (if ever). Flap “thinning” begins as edema fully resolves. Conversely, some studies suggest dependance on vascular anastomosis even 1 year after surgery (Kumar et al. 2004).

### 3.2 Timing of Pedicle Division

There are differing reports regarding the earliest time point at which pedicle division can be safely performed in patients undergoing reconstruction with free flaps. Both the type of flap and the reconstructive technique appear to be relevant. For free flaps, the literature provides only limited precise information on the time window at which successful and sufficient autonomization has been achieved (Berkane et al. 2023). For thin fasciocutaneous flaps such as the RFF, various time points have been reported. Hindocha et al. describe a case of intraoral reconstruction using an RFF in which the arterial vessel was ligated on postoperative day 10. Despite ligation and a clinically white, hard flap without capillary refill, the flap was left in situ and healed without complications or necrosis (Hindocha et al. 2013). Similarly, Godden et al. and Castling and Avery report intraorally positioned RFFs that remained vital 9 days after loss of arterial supply and showed no necrosis (Godden and Thomas 2002; Castling and Avery 2003). For flaps of different composition, such as the large‐volume LD flap, the reported time frames for successful pedicle division are also within a comparable range. Kissun et al. report a case with successful autonomization after only 6 days (Kissun et al. 2004). Other reports describe loss of arterial perfusion between 10 and 14 days after LD transfer, typically resulting in partial necrosis with survival restricted to deeper muscle portions (Khoo and Bailey 1982). For other large‐volume flaps, such as the ALT flap, case reports likewise document complete flap survival despite loss of arterial supply after 9 days (Wise et al. 2011). Taken together, the feasibility and timing of pedicle division appear to be influenced by both flap type and recipient bed, and autonomization may occur earlier in thin fasciocutaneous flaps than in larger‐volume flaps (see Sections 3.3 and 3.4).

### 3.3 Flap Types and Clinical Indications

Tissue composition, vascular contact area, and metabolic activity all influence neovascularization and the timeline to pedicle independence. In head and neck reconstruction, RFF are common for intraoral defects due to their thin, well‐vascularized skin and broad surface contact; factors that support early capillary ingrowth. While flap choice in clinical practice is still primarily guided by defect size, configuration, and the need for potential secondary procedures, advancing knowledge of flap‐specific neovascularization patterns could help predict the timing and reliability of pedicle independence. Such insights could not only improve overall free flap outcomes by tailoring flap selection to tissue and defect type but also make secondary interventions more predictable and plannable (Evans et al. 1994; Fenske et al. 2025; Chen et al. 2005). ALT flaps offer greater soft tissue volume and are commonly used for larger defects, also benefiting from favorable revascularization conditions (De Beule et al. 2016; Lakhiani et al. 2012). LD flaps, rich in muscle, promote robust angiogenesis and integrate well in extensive reconstructions (Friedrich et al. 1988; Quillen 1979; Watanabe et al. 2010). Conversely, osteocutaneous flaps such as the FFF, SFF, and DCIA are essential for bony reconstruction but exhibit slower and less reliable autonomization (Qaisi et al. 2024; Aksoyler et al. 2021). Bone tissue contributes minimally to neovascularization, leaving perfusion dependent on the surrounding soft tissue (Marenzana and Arnett 2013). Chimeric flaps, which combine multiple tissue types on a single pedicle, can improve integration by increasing vascular contact area, particularly when skin or muscle components dominate (Zhang et al. 2021; Jiang et al. 2014). However, autonomization may vary across components, and their use requires advanced planning and technical expertise. In summary, fasciocutaneous and muscle flaps tend to autonomize more quickly and predictably partly due to dense capillary networks, while bone‐containing flaps require prolonged vascular support. Recognizing these differences is essential when planning reconstructions where early pedicle independence or secondary procedures are anticipated (Sweeny et al. 2024; Cosset et al. 2024).

### 3.4 Predictors of Successful Autonomization

Flap autonomization is influenced by a combination of flap design, size, thickness, tissue type and patient‐related factors. Understanding these predictors is essential for anticipating vascular independence and guiding surgical decisions (Granzow et al. 2015; Bradshaw and Wagels 2017). Thin fasciocutaneous flaps (e.g., RFF) offer broad contact with the recipient bed, promoting early capillary ingrowth (Wong and Wei 2010; Sapino et al. 2019), whereas thick, fat‐rich, or osteocutaneous flaps have limited vascular interface and therefore slower neovascularization (Minami et al. 1989; Kearns et al. 2018). Interestingly, there is limited evidence that muscle flaps tend to integrate faster due to higher metabolic throughput and vascularity and dense perforator networks (Friedrich et al. 1988; Röjdmark et al. 2002). However, there are also lines of research that point toward faster vascular independence in fasciocutaneous flaps compared to muscle flaps. For instance, Mücke et al. evaluated 50 oral cavity free flaps and demonstrated that radial forearm flaps neovascularize fastest, whereas muscle‐containing flaps may depend on pedicles even after 12 weeks, especially in irradiated or maxillary sites (Mücke et al. 2012). Similarly, Rother et al. reviewed 23 free flaps, including fasciocutaneous and muscle transplants, and concluded no systematic advantage of muscle flaps in achieving earlier independence, but highlighted the concept of case‐dependent vascularization (Rother et al. 2020). Furthermore, recipient bed vascularity is critical for autonomization and may influence long‐term outcomes of free flap neovascularization. Here, healthy, well‐perfused beds enable robust angiogenesis, whereas irradiated, scarred, or chronically low‐grade infected tissue impairs vessel ingrowth and prolongs pedicle dependency (Tasch et al. 2022; Koesters and Chang 2023; Zirk et al. 2020; Goyal et al. 2016). Patient comorbidities—such as diabetes, vascular disease, smoking, and malnutrition—are associated with impaired microcirculation and delayed healing (Valentini et al. 2008; Ooms et al. 2023). Pharmacologic support, including vasodilators and anticoagulants, may enhance early perfusion and neovascular remodeling, though clinical evidence remains limited. Experimental agents promoting angiogenesis (e.g., Vascular Endothelial Growth Factor (VEGF)‐based therapies) are still under investigation (Zhang et al. 2015; Hosseini et al. 2022; Wang et al. 2019; Abdelfattah et al. 2020). Furthermore, preoperative patient optimization plays a critical role in supporting both anastomotic success and the subsequent autonomization of free flaps. Interdisciplinary measures—such as vascular surgical or interventional radiological procedures to improve extremity perfusion—can enhance the quality of recipient vessels and the surrounding tissue bed. By maximizing baseline blood flow and capillary density, these strategies not only reduce the risk of early ischemic failure but may also accelerate neovascularization and increase the likelihood of reliable pedicle independence. Integrating such preparatory steps into reconstructive planning could therefore improve long‐term flap viability and facilitate safer secondary interventions (Vincent et al. 2019; Kim et al. 2019; Ahn et al. 2024; Henn et al. 2018; Didzun et al. 2025). Taken together, these factors help stratify flap risk. Favorable anatomy and patient health may allow earlier interventions, while high‐risk cases, particularly thick flaps over compromised beds, require prolonged pedicle protection and cautious postoperative planning. In this context, it is important to also highlight the role of precise monitoring of flap perfusion for identifying early signs of compromised circulation and evaluating the progression toward autonomization (Knoedler, Hoch, et al. 2023). Clinical signs (e.g., skin color, capillary refill, surface temperature, pinprick bleeding) may not detect subtle perfusion deficits or early neovascular changes (Shen et al. 2021; Pafitanis and Chen 2019; Bradford 1996). As an imaging‐based add‐on, indocyanine green (ICG) angiography is widely used for intraoperative and early postoperative assessment, offering dynamic visualization of arterial inflow (Choudhary et al. 2023; Li et al. 2018; Thomas et al. 2025). Laser Doppler flowmetry measures superficial blood flow continuously and is useful for trend monitoring (Lenz et al. 2018; Kodama et al. 2025). Near‐infrared spectroscopy (NIRS) evaluates tissue oxygen saturation at varying depths, allowing insight into deeper perfusion layers (Bian et al. 2022; Thiem et al. 2021). Hyperspectral imaging is an emerging noninvasive modality that captures spatial and spectral data to assess tissue oxygenation and hemoglobin concentration, offering detailed maps of perfusion without the need for contrast agents (Kohler et al. 2021; Lindelauf et al. 2022). These tools may also help identify when a flap is beginning to establish secondary perfusion pathways, while no current method yet definitively confirms full autonomization.

### 3.5 Postoperative Complications

Impaired or delayed autonomization of free flaps can lead to a range of postoperative complications. In such cases, any disruption to the pedicle can precipitate partial or total flap necrosis. Additional complications may include wound dehiscence, delayed healing, fistula formation, persistent drainage, and susceptibility to infection due to compromised perfusion (Crawley et al. 2019; Serra et al. 2024; Smith et al. 2018). These issues often require secondary reconstructive procedures, prolonged hospitalization, and intensive wound management (Burkhard et al. 2021; Stepanovs 2016; Karamitros et al. 2025). First, in oncologic patients or those undergoing multimodal therapy (e.g., chemotherapy or radiotherapy), impaired vascularization can exacerbate tissue fragility or delay adjuvant treatments and vice versa, directly affecting oncologic outcomes (Chang et al. 2013; Fracol et al. 2016). Second, in high‐risk patient populations—such as smokers, diabetics, or individuals with peripheral vascular disease—baseline microcirculatory deficits further impair neovascularization, compounding the risk of flap failure (Kallio et al. 2015; Rosado et al. 2015). Flaps with limited neovascular integration are especially vulnerable during interventions near the vascular axis, such as lymphadenectomy or hardware removal, where even minor trauma or inflammation can impair perfusion. The unpredictability of the autonomization timeline adds further complexity, underscoring the need for cautious postoperative planning and reliable monitoring to mitigate avoidable complications (Mücke et al. 2012; Mueller et al. 2017). Here, postoperative strategies may also contribute to promoting flap autonomization by stimulating neovascularization and adaptive perfusion pathways. Techniques such as flap “training”—for example, progressive dangling protocols or controlled compression—expose the flap to graded ischemic stress, encouraging vascular remodeling and enhancing tolerance to reduced pedicle dependency. Similarly, ischemic pre‐ or postconditioning protocols, recently described in the reconstructive literature, aim to trigger protective molecular cascades and microvascular adaptations that support secondary perfusion. While clinical evidence remains limited and heterogeneous, these approaches highlight the potential of targeted postoperative interventions to accelerate randomization, stabilize flap physiology, and expand the window for safe secondary procedures (Min et al. 2025; Krijgh et al. 2020).

### 3.6 Unsolved Research and Clinical Questions

Establishing standardized indicators of vascular independence would improve consistency in clinical decision‐making and allow for more personalized postoperative care. Enhanced monitoring techniques—such as Doppler ultrasound, near‐infrared spectroscopy, and hyperspectral imaging—may not only detect early perfusion deficits but also offer valuable insights into the progression of neovascularization. Real‐time assessment of perfusion dynamics could support evidence‐based timing for flap manipulation, reduce reliance on empirical judgment, and optimize resource allocation (Zhang et al. 2021; Choudhary et al. 2023; Thomas et al. 2025). Another unresolved question is how autonomization varies across different flap types and recipient sites, and whether tissue composition or anatomical region significantly alters the revascularization process. At last, it remains unclear to what extent patient‐specific factors—such as comorbidities, smoking status, or prior radiation—modulate the timeline or success of flap autonomization (Copelli et al. 2017; Kwasnicki et al. 2021; Smit et al. 2010; Ouyang et al. 2024; Corbitt et al. 2014; Shimbo et al. 2023; Rocans et al. 2024; Nguyen et al. 2013).

## 4 Discussion

Autonomization of microvascular free flaps remains a poorly defined process with direct implications for postoperative decision‐making. While early neovascularization has been observed within the first postoperative week, the lack of objective markers to confirm flap independence continues to limit surgical confidence, particularly when secondary procedures are required. Current evidence highlights wide variability based on flap type, tissue composition, and recipient bed quality (Fenske et al. 2025; Chen et al. 2005; Aksoyler et al. 2021; Cosset et al. 2024; Koepple et al. 2019). Therefore, this review aims to synthesize these findings and emphasize the need for standardized criteria to safely guide intervention timing and improve reconstructive outcomes.

**FIGURE 1:**
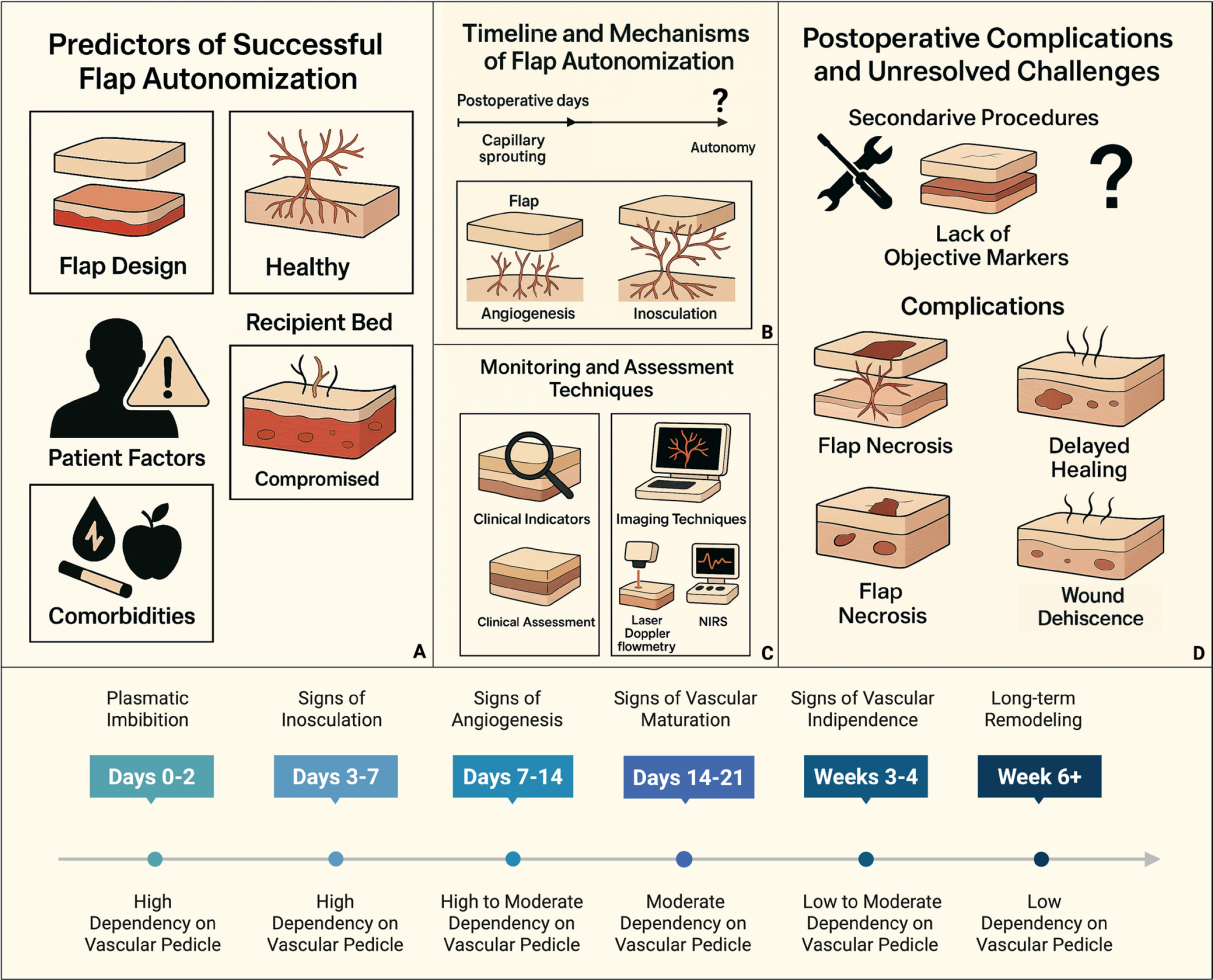
Overview of the biological mechanisms, clinical determinants, and challenges related to flap autonomization in microvascular reconstructive surgery. (A) Predictors of successful flap autonomization: key anatomical and physiological factors including flap design, recipient bed condition, patient‐specific variables, and comorbidities influence neovascularization and timing of pedicle independence. (B) Timeline and mechanisms of flap autonomization: capillary sprouting, inosculation, and subsequent integration with the recipient bed are described in the literature as key steps in flap autonomization, but the precise timeline remains variable and not yet fully understood. (C) Monitoring and assessment techniques: flap viability is monitored through clinical indicators (e.g., capillary refill, bleeding) and objective tools such as indocyanine green (ICG) angiography, laser Doppler flowmetry, and near‐infrared spectroscopy (NIRS). (D) Postoperative complications and unresolved challenges: the absence of validated markers for vascular independence limits the timing of secondary procedures. Premature intervention may result in necrosis, delayed healing, or flap loss. In the bottom half of the figure a timeline depicts key time frames and landmarks of free flap autonomization: plasmatic imbibition (Nelissen and White 2014; Smahel 1971; Stell 1977); inosculation (Yoon and Jones 2016; Angelos et al. 2010; Tsur et al. 1980; Foerster et al. 2022); angiogenesis (neovascularization) (Godden and Thomas 2002; Wise et al. 2011; Burns et al. 2005; Yoon et al. 2018; Lucas 2017); vascular maturation (Berkane et al. 2023; Alsina‐Rius et al. 2022; Garutti et al. 2024); autonomization (Granzow et al. 2015; Mücke et al. 2012; Giordano et al. 2022); long‐term remodeling (Bagheri et al. 2024; Ludolph et al. 2019; Thariat et al. 2024; Clarke and Chen 1992).

Overall, our review found that successful autonomization of microvascular free flaps is governed by a complex interplay of flap type, recipient bed quality, and patient‐specific factors. Fasciocutaneous and muscle‐based flaps show earlier and more predictable neovascular integration, whereas osteocutaneous flaps tend to remain pedicle‐dependent longer. Despite advances in imaging and monitoring, the absence of objective “gold standard” markers for vascular independence remains a major clinical limitation, underscoring the need for standardized criteria to safely guide postoperative interventions and minimize flap‐related complications (Figure 1).

In the literature, free flaps are consistently highlighted as a cornerstone across reconstructive fields, providing reliable options for complex defects where local or regional tissue is insufficient (Knoedler, Hoch, et al. 2024; Chen et al. 2005; Cosset et al. 2024; Kearns et al. 2018; Copelli et al. 2017; Corbitt et al. 2014; Knoedler, Kauke‐Navarro, et al. 2025; Balasubramanian et al. 2012). Reliable perfusion and early vascular stability are critical for long‐term outcomes (Kearns et al. 2018). Monitoring is also more challenging intraorally, which elevates the importance of flap choice and meticulous surgical planning (Yoshino et al. 1996; Nielsen et al. 2011). Furthermore, the confined surgical field, risk of vessel compression from postoperative edema, and limited options for re‐anastomosis in salvage scenarios heighten the consequences of even minor ischemic events. Secondary procedures, such as tracheostomy closure or hardware removal, often occur close to the pedicle and are therefore more likely to jeopardize flap integrity if performed prematurely (Khoshnevis et al. 2018; Katira et al. 2019; Mousavi et al. 2019). These risks underscore the need for reliable markers of autonomization to better inform surgical timing.

The reported time frames for a possible pedicle division are relatively short for local rotation flaps such as the paramedian forehead flap. Rudy et al. describe autonomization as early as postoperative day 7, allowing for early pedicle division with this technique (Rudy et al. 2021). However, these findings cannot be directly translated to free flaps, as they differ in composition, volume, and vascular supply. Among free flaps, fasciocutaneous flaps such as the radial forearm flap (RFF) (Kristensen et al. 2017) are comparatively well studied (Berkane et al. 2023). Even in this context, however, recommendations for the earliest safe timing of pedicle division are largely based on individual case reports. For the RFF, the literature reports successful pedicle division as early as the 6th postoperative day, with more frequent reports of division around postoperative day 9 with complete flap survival (Hindocha et al. 2013; Godden and Thomas 2002; Castling and Avery 2003; Burns et al. 2005). For larger‐volume flaps such as the latissimus dorsi (LD) and anterolateral thigh (ALT) flaps, the reported time points vary. There is at least one case report of LD flap survival after pedicle division on Day 6 (Kissun et al. 2004), while other reports suggest that a longer period is required to achieve full autonomization (Khoo and Bailey 1982; Wise et al. 2011). Otherwise, partial necrosis with survival of the deeper muscle parts might follow (Khoo and Bailey 1982). In all cases, the complex process of flap autonomization, the specific flap type used, patient‐related factors, and the characteristics of the recipient site (flap bed) must be taken into account.

Extremity and breast reconstruction often prioritize structural durability, volume restoration, and aesthetic contouring, with relatively more accessible monitoring (Koepple et al. 2019; Agaoglu and Erol 2009; Knoedler, Kauke‐Navarro, et al. 2023, 2024; Kwok et al. 2018; Capek et al. 2018; Arakelyan et al. 2022). For instance, in lower limb salvage procedures, bone stability and weight‐bearing capacity are prioritized, while in breast reconstruction, symmetry and soft tissue pliability dominate the decision‐making process. In these contexts, longer autonomization timelines may be more acceptable, and external signs of perfusion are more easily monitored (Rozen et al. 2010; Tiongco et al. 2025). Additionally, the availability of external adjuncts—such as vacuum‐assisted closure, external fixators, or pressure‐offloading systems—can support flap viability and reduce complications, advantages less applicable in head and neck contexts (Salgado et al. 2010; Patel et al. 2017). Experimental research into flap autonomization is most active in animal models and tissue engineering studies. Rodent and porcine models have demonstrated that angiogenic growth factors, stem cell therapies, and pre‐vascularized scaffolds can accelerate revascularization and reduce pedicle dependency. Among these, bioengineered constructs, comprising biodegradable scaffolds seeded with endothelial or progenitor cells, oftentimes incorporating 3D‐printed vascular channels, have emerged as a particularly promising strategy (Berkane et al. 2023; Zhang et al. 2015; Baecher et al. 2023; Foroglou et al. 2016; Masson‐Meyers et al. 2023). Some constructs also include controlled‐release systems for VEGF or other pro‐angiogenic factors to enhance early inosculation, while co‐culture models with pericytes or preconditioning techniques using arteriovenous loops aim to further improve vessel maturation (Marchesini et al. 2020; Jagasia et al. 2025). These innovations have demonstrated functional perfusion in preclinical settings and could potentially shorten the timeline to flap independence, particularly in high‐risk or irradiated recipient beds. However, translation to human surgery remains limited by concerns over immune rejection, inconsistent integration, production costs, and regulatory barriers (Shandalov et al. 2014; Egro et al. 2024; Schrey et al. 2010). Clinical challenges such as graft thrombosis and scaffold degradation further complicate their use. Nevertheless, studies have proposed hybrid approaches in which bioengineered constructs are used to supplement traditional flaps during staged reconstruction (Chen et al. 2005, 2007; Zhang et al. 2015). When combined with intraoperative perfusion monitoring, such strategies may allow tailored interventions for patients at high risk of pedicle compromise. Overall, further investigation into the mechanisms and timeline of free flap vascular autonomization could enable safer planning of secondary interventions and reduce the risk of flap loss. Establishing standardized indicators of vascular independence would improve consistency in clinical decision‐making and allow for more personalized postoperative care. Enhanced monitoring techniques—such as Doppler ultrasound, near‐infrared spectroscopy, and hyperspectral imaging—may not only detect early perfusion deficits but also offer valuable insights into the progression of neovascularization. Real‐time assessment of perfusion dynamics could support evidence‐based timing for flap manipulation, reduce reliance on empirical judgment, and optimize resource allocation (Copelli et al. 2017; Kwasnicki et al. 2021; Smit et al. 2010; Ouyang et al. 2024; Corbitt et al. 2014; Shimbo et al. 2023; Rocans et al. 2024; Nguyen et al. 2013).

In summary, this review underscores the critical role of autonomization in microvascular free flap surgery to enable the long‐term safety of microvascular reconstructions for both patients and surgeons. This is particularly important in the head and neck region, where complex anatomy, prior treatment history, and functional demands often increase the risk of vascular compromise. For clinicians, the findings suggest that careful flap selection, personalized surgical planning, and cautious timing of secondary procedures could reduce complications, especially in high‐risk cases. While traditional approaches still dominate practice, new tools such as intraoperative perfusion imaging or pharmacologic angiogenesis support might enhance decision‐making. Experimental innovations, like bioengineered vascular scaffolds or VEGF‐enhanced constructs, may eventually support earlier flap independence, though further validation is needed before they can be adopted into routine care. Importantly, there is currently no gold standard for assessing flap autonomization, and clinical decisions still rely heavily on empirical timing and subjective indicators. A meaningful shift toward more individualized, evidence‐based strategies will require validated biomarkers, standardized evaluation protocols, and long‐term outcome data across diverse clinical scenarios. Patients should be aware that staged procedures might be necessary and that the timing of follow‐up surgeries could impact flap health. As research progresses, future treatments may allow for faster healing, fewer surgical delays, and more predictable outcomes, particularly in challenging reconstructions. Ultimately, close communication between patients and care teams will remain essential, while ongoing innovation may gradually broaden and improve reconstructive options.

## 5 Limitations

While this narrative review offers a comprehensive synthesis of the current understanding of flap autonomization in microvascular reconstructive surgery, several limitations should be considered. First, as a narrative rather than a systematic review, the methodology lacks quantitative meta‐analysis and may be subject to selection bias, despite efforts to maintain rigor and thematic consistency. Second, the heterogeneity of the included studies—in terms of flap types, anatomical sites, monitoring techniques, and outcome definitions—limits the ability to draw standardized or generalizable conclusions regarding optimal timelines for pedicle independence. Additionally, much of the evidence on autonomization timing and neovascularization mechanisms stems from case reports, small cohort studies, or preclinical animal models, making extrapolation challenging. Moreover, the lack of standardized criteria for confirming vascular independence—such as validated biomarkers or perfusion thresholds—continues to hinder objective assessment. Existing data are limited by small sample sizes, short follow‐up periods, and inconsistent reporting standards, underscoring the need for high‐quality, multicenter trials to validate their utility in real‐world settings. Importantly, free flap surgery is a multidisciplinary field spanning multiple specialties—including plastic surgery, maxillofacial surgery, and surgical oncology—with diverse clinical goals and protocols. Taken together, these limitations highlight the need for further research to establish evidence‐based guidelines for flap autonomization, particularly in high‐risk anatomical regions such as the head and neck.

## 6 Conclusion

Flap autonomization represents a pivotal yet incompletely understood process in microvascular reconstructive surgery, with direct implications for postoperative decision‐making, flap monitoring, and the safe timing of secondary interventions. This review highlights that while early neovascularization can begin within the first postoperative week, the timing and consistency of complete vascular independence vary widely by flap type, recipient bed condition, and patient factors. Fasciocutaneous and muscle‐based flaps generally demonstrate faster and more predictable autonomization than osteocutaneous constructs. Despite advancements in imaging and microsurgical technique, the absence of reliable intraoperative or postoperative markers continues to necessitate conservative clinical protocols. Experimental strategies—including bioengineered vascular scaffolds, pro‐angiogenic therapies, and real‐time perfusion assessment tools—offer potential to refine flap management and reduce complication risks. However, these innovations require further validation before routine clinical adoption. Future research should focus on standardizing outcome metrics, validating objective markers of vascular independence, and bridging experimental advances with clinical realities to enhance both flap survival and patient safety in complex reconstructions.

## Funding

Open Access funding enabled and organized by Projekt DEAL. No further funding was provided for this study.

## Ethics Statement

The authors have nothing to report.

## Conflicts of Interest

The authors declare no conflicts of interest.

## Data Availability

Data sharing not applicable to this article as no datasets were generated or analysed during the current study.
